# *QuickStats*: Age-Adjusted Rates for Homicides,* by Race/Ethnicity^†^— United States, 1999–2015

**DOI:** 10.15585/mmwr.mm6631a9

**Published:** 2017-08-11

**Figure:**
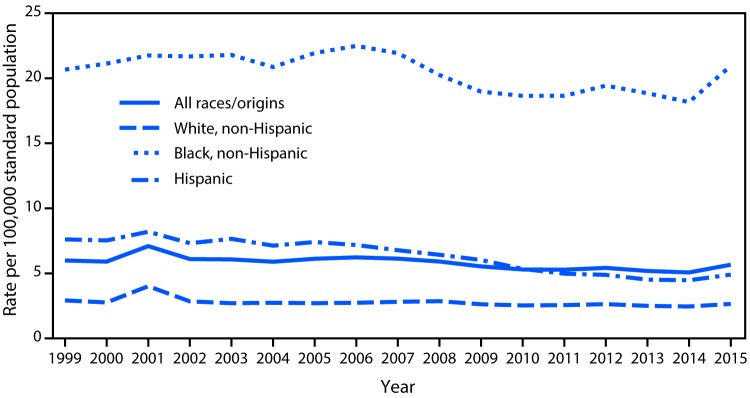
During 1999–2014, a general decline in homicide trends for non-Hispanic white, non-Hispanic black, and Hispanic populations occurred, followed by a significant increase in the rates for all three groups between 2014 and 2015. In 2015, homicide rates were 5.7 deaths per 100,000 for the total population, 20.9 for non-Hispanic blacks, 4.9 for Hispanics, and 2.6 for non-Hispanic whites. During 1999–2015, rates of deaths from homicide were highest for non-Hispanic blacks and lowest for non-Hispanic whites and declined the most for Hispanics.

**Source:** CDC/National Center for Health Statistics, National Vital Statistics System, 1999–2015, Mortality. CDC Wonder online database. https://wonder.cdc.gov/ucd-icd10.html.

For more information on this topic, CDC recommends the following link: https://www.cdc.gov/violenceprevention/index.html.

